# Xanthohumol ameliorates cardiac injury induced by sepsis in a mice model: role of toll-like receptor 4

**DOI:** 10.25122/jml-2023-0016

**Published:** 2023-07

**Authors:** Sarah Mohammed Hussain Hadi, Sahar Majeed, Fadhaa Abdulameer Ghafil, Kaswer Altoraihi, Najah Rayish Hadi

**Affiliations:** 1 Department of Pharmacology and Therapeutics, Faculty of Medicine, University of Kufa, Najaf, Iraq; 2 Medical College, Department of Pharmacology and Therapeutics, University of Kufa, Najaf, Iraq

**Keywords:** sepsis, CLP, TLR- 4/NF- kB signaling pathways, Xanthohumol, ANOVA: Analysis Of Variance, Bcl-2: B-cell lymphoma 2, C°: Celsius Degree, CLP: Cecal Ligation And Puncture, cTn-I: cardiac troponin I, CK-MB: creatinine kinase MB, DMSO: Dimethyl Sulfoxide, ELISA: enzyme-linked immunosorbent assay, ERK: Extracellular signal-regulated kinase, JNK: c-Jun N-terminal kinase, IL-6: Interleukin 6, IL-10: Interleukin 10, IP: intraperitoneally, LPS: lipopolysaccharide, KEAP-1: Kelch-like ECH-associated protein 1, MAPK: Mitogen-Activated Protein Kinase, MD-2: Myeloid differentiation factor-2, MIF: Macrophage migration inhibitory, NF-κB: Nuclear factor kappa-light-chain-enhancer of activated B cells, Nrf2: nuclear factor erythroid 2-related factor 2, ROS: reactive oxygen species, TNF- α: Tumor necrosis factor alpha, TLR4: Toll-like receptor 4, XN: Xanthohumol

## Abstract

Sepsis, a life-threatening condition arising from infection, often results in multi-organ failure, including cardiac dysfunction. This study investigated Xanthohumol, a natural compound, and its potential mechanism of action to enhance heart function following sepsis. A total of twenty-four adult male Swiss albino mice were allocated randomly to one of four equal groups (n=6): sham, CLP, vehicle Xanthohumol the same amount of DMSO injected IP 10 minutes before the CLP, and Xanthohumol group (0.4 mg/kg of Xanthohumol administered IP before the CLP process). Toll-like receptor 4, pro-inflammatory mediators, anti-inflammatory markers, oxidative stress indicators, apoptosis markers, and serum cardiac damage biomarkers were measured in the cardiac tissue using ELISA. Data with normal distribution were analyzed using t-test and ANOVA tests (p<0.05). In comparison to the sham group, the sepsis group had significantly higher levels of TLR-4, IL-6, TNF-α, MIF, F2-isoprostane, caspase-3, cTn-I, and CK-MB, while the pre-treated group with Xanthohumol had significantly lower levels (p<0.05) of these markers than the sepsis group. Bcl-2 showed no significant difference in Xanthohumol pre-treated group relative to the sepsis group, while IL-10 was significantly elevated. Xanthohumol dramatically reduced cardiac tissue injury (p<0.05) relative to the CLP group. By blocking the downstream signal transduction pathways of TLR-4 and NF-kB, Xanthohumol was shown to lessen cardiac damage in male mice during CLP-induced polymicrobial sepsis.

## INTRODUCTION

The term “sepsis” has lately been described as systemic inflammatory response syndrome (SIRS), which develops as a result of infection and leads to organ failure [1]. Diagnostic criteria for sepsis include the presence of infection accompanied by altered mental status, leukocytosis (WBC>12,000/mL) or leukopenia (WBC<4,000/mL), tachycardia (heart rate>90 beats per minute), abnormal body temperature (T>38°C or <36°C), elevated respiratory rate (>20 breaths per minute), decreased partial pressure of CO2 (PaCO2<32 mmHg), or alterations in any of these parameters [2].

When pathogen-associated molecular patterns (PAMPs) and danger-associated molecular patterns (DAMPs) connect to pattern recognition receptors during sepsis, several pathways lead to the development of organ failure (PRRs), such as retinoic acid-inducible gene 1 (RIG-1-like receptors), toll-like receptors (TLR), nucleotide-binding oligomerization domain (NOD-like receptors), C-type lectin receptors (CLRs), and NOD-like receptors, which are found on the surfaces of immune cells and cardiomyocytes. As a result, proinflammatory cytokines, including TNF, IL-1, and IL-6, are produced and released as a result of the activation of intracellular signal transduction pathways [3, 4]. These cytokines serve as essential indicators for disease identification, assessment, and illness management [5].

Toll-like receptor 4 (TLR4) was the first human toll receptor identified and is found on both the surface of cardiomyocytes and immune cells. TLR4 has been associated with various cardiac conditions, including myocardial infarction (MI), ischemia-reperfusion injury (I/R damage), heart failure, myocarditis, aortic valve disease, atherosclerosis, hypertension, and myocardial inflammation [6]. Sepsis may cause heart dysfunction, a severe illness known as septic cardiomyopathy (SCM) [7]. There has been a long-running effort to find new antibacterial agents. The growing drug resistance, nevertheless, has outpaced the development of new medications. The restricted availability of screening libraries is one of the main obstacles. The development of these chemical libraries may be aided by natural plant products like chalcones [8]. One such compound is Xanthohumol (XN), a prenylated chalcone extracted from the Hops plant (Humulus lupulus) [9]. Several studies on the anti-inflammatory, antioxidant, hypoglycemic, anticancer, and other properties of XN in vitro and in vivo clearly indicate a potential for the prevention and treatment of many illnesses, including sepsis [8]. Therefore, this study aimed to investigate the effects of Xanthohumol on sepsis-induced SCM.

## MATERIAL AND METHODS

### Study location and design

The study was conducted at the Middle Euphrates Unit for Cancer Research and the Department of Pharmacology and Therapeutics, Faculty of Medicine, University of Kufa. The study population comprised four groups, each comprising 6 adult male Swiss albino mice weighing between 20 and 30g. Group 1 served as the negative control (sham group), where mice were anesthetized and underwent laparotomy surgery without exposure to cecal ligation and puncture (CLP) operation. Group 2 was the positive control (CLP group), where all mice were subjected to the CLP operation to induce sepsis [10]. Group 3 (Vehicle Xanthohumol group) received the same volume of DMSO intraperitoneally 10 minutes before the CLP operation. The final group, Group 4 (Xanthohumol group), was administered 0.4 mg/kg of Xanthohumol intraperitoneally 10 minutes before the CLP operation. The dose was selected based on a previous similar study [11]. Mouse mortality was monitored, and the mice were observed for 24 hours.

### Experimental procedure

The mice were anesthetized with 10 mg of Xylazine and 100 mg/kg of ketamine intraperitoneally before surgery. The cecum was then made visible by an abdominal laparotomy with a 1.5 cm midline incision. After that, a G-21 needle was used to penetrate the cecum two times. The puncture holes were gently pushed through with a modest amount of fecal material. After being poked and knotted, the cecum was reinserted into the abdominal cavity. The abdomen was then closed with a 3.0 surgical suture, and 1 ml of saline solution was injected subcutaneously. The mice were kept on their backs until awakening and then returned to their cages [12].

### Preparation of drug

Xanthohumol (XN) at a dosage of 0.4 mg/kg was administered intraperitoneally 10 minutes before the CLP procedure using XN purchased from ChemScene and diluted in DMSO [11].

### Preparation of samples

#### Blood samples

Mice were anesthetized to a terminal state, and blood samples were collected by puncturing their hearts with a suitable needle. The blood was drawn into gel tubes and left to settle at room temperature for 10–20 minutes before being centrifuged at 2000 RPM for 20 minutes at 4°C. The supernatant, free from sediment, was cautiously collected for analysis. The blood samples were used to measure cardiac troponin I (cTn-I) and creatinine kinase MB (CK-MB) levels, vital markers for assessing cardiac tissue damage [13].

#### Tissue samples

Upon collection, tissue samples from the mice's hearts were divided into two halves. One half was immediately fixed in 10% neutral formalin for histopathological analysis, while the other half was frozen for subsequent ELISA analysis.

### Histopathological examination

A microtome was used to manually cut 4-5 m thick paraffin slices into pieces after being fixed in paraffin. After dewaxing, the components were stained with hematoxylin and eosin (H&E) [14]. Each heart section (n=5 sections per heart) was inspected under an optical microscope to assess the degree of heart damage and take pictures. To roughly assess the variance in heart damage, histological sections from all groups were analyzed and graded using Zingarelli's technique [15]. This scoring system consisted of four distinct severity levels [16, 17]:

Score 0: represents normal tissue

Score 1: represents mild interstitial edema and localized necrosis

Score 2: represents moderate myocardial cell swelling and diffused necrosis

Score 3: represents severe ischemia and neutrophil buildup

Score 4: represents extremely severe with contraction bands, leukocyte infiltration, ischemia, and hemorrhage.

### ELISA analysis

Myocardial tissues were homogenized in phosphate-buffered saline (PBS) 1:10 W/V containing 0.5% Triton X100 with a protease inhibitor cocktail after being rinsed in ice-cold saline to remove any remaining red cells or clots. The resulting homogenates were centrifuged at 4°C for 20 minutes at 3000 rpm. The supernatant was used to measure myocardial tissue levels of MIF, TNF-α, IL-10, IL-6, caspase-3, F2 isoprostane, Bcl-2, and TLR-4, using commercial ELISA kits.

### Statistical analysis

Statistical analysis was done using SPSS version 26 software (IBM Corp., Armonk, NY, USA). To assess the normal distribution of the data, the Kolmogorov-Smirnov and Shapiro tests were performed. For normally distributed data, t-test and ANOVA tests were conducted with a significance threshold of 0.05 (parametric data) [18].

### The mortality rate of mice

The mortality rate of mice in the experimental study was reported based on six mice per group. The mortality rate was 50% after CLP without treatment and 16.6% after treatment with Xanthohumol.

## RESULTS

**Figure 1:**
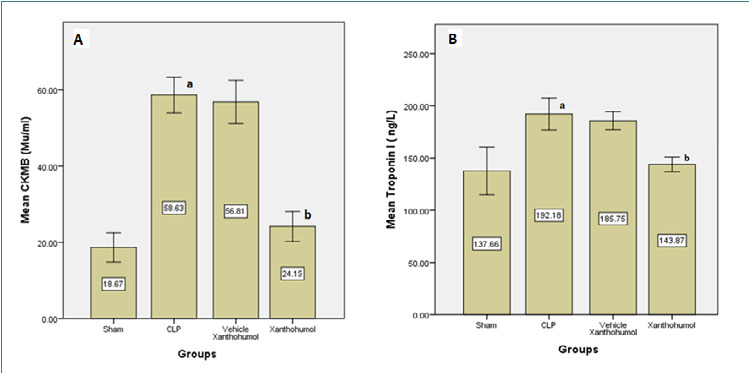
Mean differences in Ck-MB (A) and cardiac troponin I (cTn-I) (B) measured in the serum of the four experimental groups, (a) significant difference in CLP group with the sham group (p<0.05), (b) significant difference in the Xanthohumol group with CLP group (p<0.05)

**Figure 2:**
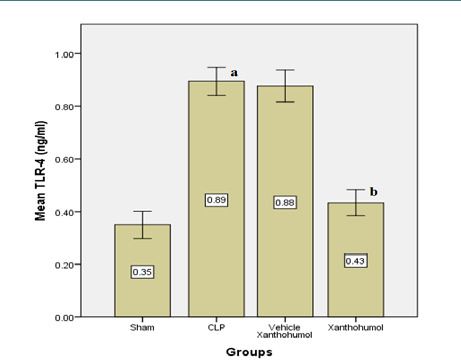
Mean TLR-4 levels measured in the cardiac tissue of the four experimental groups, (a) significant difference in the CLP group with the sham group (p<0.05), (b) significant difference in the Xanthohumol group with the CLP group (p<0.05)

**Figure 3:**
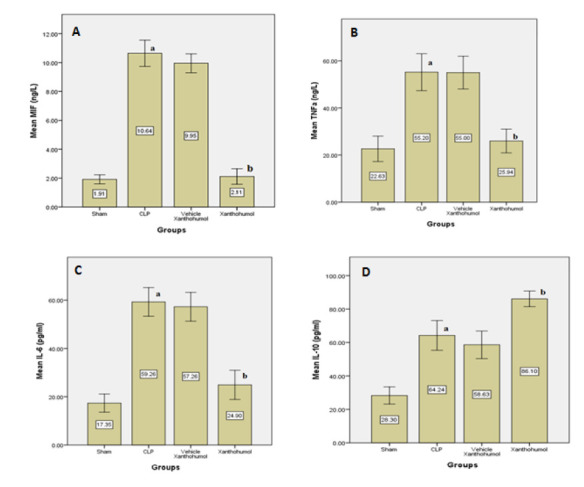
Mean levels of inflammatory markers in cardiac tissue of the four experimental groups. A: MIF, B: TNF-a, C: IL-6, and D: IL-10. (a) significant difference in the CLP group with sham group (p<0.05), (b) significant difference in the Xanthohumol group with the CLP group (p<0.05)

**Figure 4:**
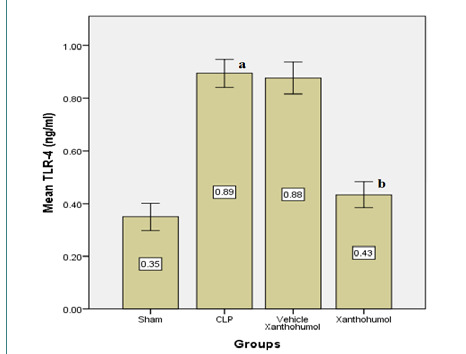
Mean levels of F2-isoprostane measured in the cardiac tissue of the four experimental groups, (a) significant difference in the CLP group with the sham group (p<0.05), (b) significant difference in the Xanthohumol group with the CLP group (p<0.05)

**Figure 5:**
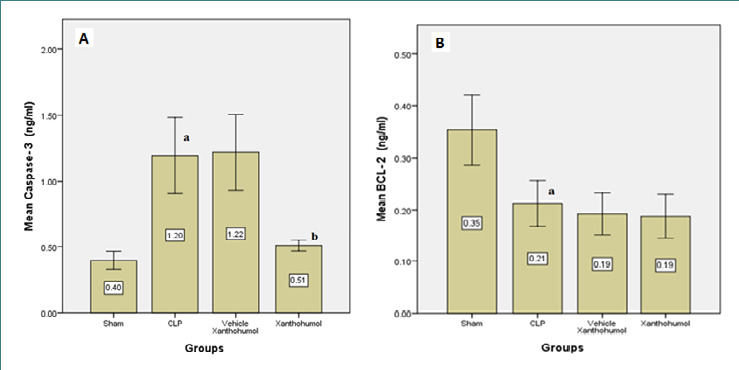
Mean caspase-3 (A) and Bcl-2 (B) levels measured in the cardiac tissue of the four experimental groups. (a) significant difference in the CLP group with the sham group (p<0.05), (b) significant difference in the Xanthohumol group with the CLP group (p<0.05)

Blood levels of cardiac troponin I (cTn-I) and creatinine kinase MB (CK-MB), indicators of myocardial damage, were significantly reduced in the Xanthohumol group compared to the CLP group (Figure 1 A-B). Furthermore, Xanthohumol effectively lowered the expression of TLR-4 in cardiac tissue (Figure 2) and reduced the levels of pro-inflammatory cytokines, including IL-6, MIF, and TNF-α, as shown in Figure 3 A-D. In contrast, the anti-inflammatory cytokine IL-10 was significantly increased in the Xanthohumol group, indicating a decrease in cardiac tissue inflammation. Moroever, Xanthohumol decreased the oxidative stress marker F2-isoprostane (Figure 4), and the apoptosis rate by lowering the pro-apoptotic caspase-3 with no impact on the anti-apoptotic Bcl-2 (Figure 5 A-B).

### Histological findings

**Figure 6:**
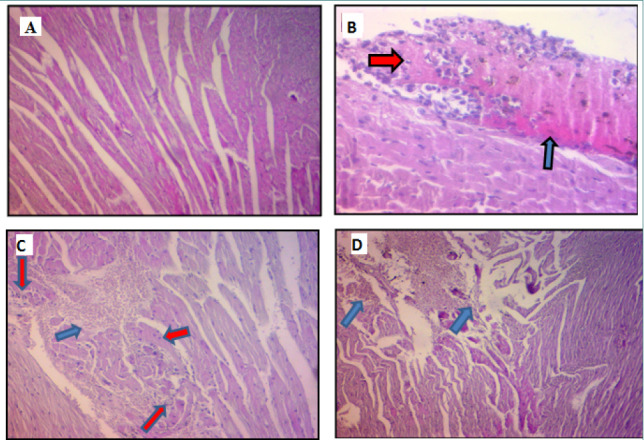
Histopathological examination of myocardial tissue. A: Sham group: normal looking cardiac tissue; B: CLP group: score 4, damaged myocardial tissue; C: Vehicle Xanthohumol group: score 4, damaged myocardial tissue; D: Xanthohumol group, score 2, moderate. These sections were stained with H&E (40X); blue arrows represent areas with hemorrhage, while red arrows show areas with acute inflammatory cells

Histological examination of the myocardial tissue revealed distinct histopathological characteristics among the experimental groups. The sham group had normal histological results. The CLP group and the vehicle Xanthohumol group revealed significant myocardial damage with the emergence of contraction bands, polymorph nuclear leukocytes (PMN) infiltration, interstitial edema, and localized extravasation of red blood cells (score 4). However, the Xanthohumol group was slightly different from the sham group, which showed mild to moderate architectural modifications (score 2). Histological findings are seen in Figure 6 A-D.

## DISCUSSION

As a result of an unbalanced immune response to infectious microorganisms, sepsis, also known as a systemic inflammatory response syndrome that develops during infection, leads to multiorgan dysfunction, including cardiac dysfunction [19]. The myocardial dysfunction in sepsis is influenced by Toll-like receptor 4 (TLR4)-mediated signaling pathways, which induce inflammation and the generation of reactive oxygen species (ROS) [20]. Before TLR4 activation, LPS binds to the CD14 protein, which then transfers LPS to the TLR4/MD-2 complex, causing the release of pro-inflammatory cytokines through TRIF- and MyD88-dependent pathways. The malfunction of the myocardium induced by sepsis involves an important signal transduction mechanism linked to Mitogen-Activated Protein Kinases (MAPKs). Stimulation of cardiomyocytes with LPS leads to increased activation of ERK, JNK, and p38 MAPK pathways [21].

Toll-like receptor-4 (TLR-4) levels in cardiac tissue were measured using an enzyme-linked immunosorbent assay (ELISA), and they were found to be higher in the CLP group. In contrast, the expression of TLR-4 was inhibited in the Xanthohumol pretreated group, which may indicate that some of the anti-inflammatory effects of Xanthohumol are caused by the antagonist activity on the TLR4/MD-2 complex that prevents NF-κB activity [22]. As Xanthohumol blocks the NF-κB and Akt pathways that are triggered by LPS, the blood protein levels of cardiac troponin I (cTnI) and creatinine kinase MB (CK-MB) also significantly decreased in comparison to the CLP group [23]. Inhibition of the NF-κB pathway has been associated with the prevention of various cardiovascular diseases, including hypertension, myocardial infarction, and arteriosclerosis [23].

Xanthohumol treatment also decreased tissue levels of pro-inflammatory cytokines (IL-6, TNF-α, and MIF) and increased levels of the anti-inflammatory cytokine IL-10 in the cardiac tissue compared to the CLP group. This effect is likely mediated by the downregulation of TLR4 and myeloid differentiation protein 2 (MD2), suppressing NF-κB signaling and downstream inflammatory cytokines. Additionally, Xanthohumol's activation of the Keap1-Nrf2 pathway may reduce F2-isoprostane, suppressing lipid peroxidation and preserving antioxidant enzyme activity, protecting against oxidative damage and limiting ROS generation [8, 24-27]. Moreover, Xanthohumol may protect the myocardium against apoptosis by inhibiting caspase-3. The exact mechanism responsible for caspase-3 downregulation remains uncertain, but it may be related to Xanthohumol's antagonist impact on TLR-4, leading to NF-κB suppression or inhibition of the MAPK/ERK pathway, which ultimately lowers inflammatory cytokines such as TNF-α. Increased transcription and translation of inflammatory cytokines during sepsis have been implicated in apoptosis [28]. Interestingly, Bcl-2 levels remained unchanged in our study, and further investigations are required to elucidate the underlying reasons for this finding.

## CONCLUSION

The findings of the study support the hypothesis that Xanthohumol has significant anti-inflammatory, anti-oxidative, and anti-apoptotic properties and effectively mitigates cardiac damage induced by sepsis.
